# In-field stereotactic body radiotherapy (SBRT) reirradiation for pulmonary malignancies as a multicentre analysis of the German Society of Radiation Oncology (DEGRO)

**DOI:** 10.1038/s41598-021-83210-3

**Published:** 2021-02-25

**Authors:** Caroline John, Riccardo Dal Bello, Nicolaus Andratschke, Matthias Guckenberger, Judit Boda-Heggemann, Eleni Gkika, Frederick Mantel, Hanno M. Specht, Carmen Stromberger, Franz Zehentmayr, Oliver Blanck, Panagiotis Balermpas

**Affiliations:** 1 Department of Radiation Oncology, University Hospital Zürich, University of Zurich, Zurich, Switzerland; 2 Department of Radiation Oncology, Universitätsmedizin Mannheim, Medical Faculty Mannheim, Heidelberg University, Mannheim, Germany; 3 Department of Radiation Oncology, University Medical Center Freiburg, Freiburg, Germany; 4 Department of Radiation Oncology, University Hospital Würzburg, Würzburg, Germany; 5 Clinic for Radiation Oncology Freising, Freising, Germany; 6 Department of Radiation Oncology, Charite – Universitätsmedizin Berlin, Corporate Member of Freie Universität Berlin, Humboldt-Universität Zu Berlin, and Berlin Institute of Health, Berlin, Germany; 7 Department of Radiation Oncology, Paracelsus Medical University, Salzburg, Austria; 8 Department of Radiation Oncology, University Medical Center Schleswig-Holstein, Kiel, Germany; 9 Department of Radiation Oncology, University of Zurich, Rämistrasse 100, 8091 Zurich, Switzerland

**Keywords:** Radiotherapy, Non-small-cell lung cancer

## Abstract

Data of thoracic in-field reirradiation with two courses of stereotactic body radiotherapy (SBRT) is scarce. Aim of this study is to investigate feasibility and safety of this approach. Patients with a second course of thoracic SBRT and planning target volume (PTV) overlap were analyzed in this retrospective, multicenter study. All plans and clinical data were centrally collected. 27 patients from 8 centers have been amenable for evaluation: 12 with non-small-cell lung cancer, 16 with metastases, treated from 2009 (oldest first course) to 2020 (latest second course). A median dose of 38.5 Gy to the 65%-isodose over a median of 5 fractions was prescribed in the first course and 40 Gy in 5 fractions for the second SBRT-course. Median PTV of the second SBRT was 29.5 cm^3^, median PTV overlap 22 cm^3^. With a median interval of 20.2 months between the two SBRT-courses, 1-year OS, and -LCR were 78.3% and 70.3% respectively. 3 patients developed grade 1 and one grade 2 pneumonitis. No grade > 2 toxicity was observed. Peripheral location and dose were the only factors correlating with tumor control. A second SBRT-course with PTV overlap appears safe and achieves reasonable local control.

## Introduction

Stereotactic body radiotherapy (SBRT) has evolved into an effective and safe treatment modality for both primary^1^ and secondary^2^ pulmonary malignancies. An improved efficacy^3^ and reduced toxicity^4^ compared to conventionally fractionated regimens, as well as the lower rate of complications compared to lobectomy^1^ are the main advantages of this treatment technique. These advantages led to the establishment of SBRT as standard-of-care even for medically inoperable patients^5^. Although most patients will experience long-time local control and/or distant failure, there is a considerable percentage of 7–20%, depending on histology, localization and dose/regimen used, that will eventually experience recurrence in the previously irradiated region^5–8^. Salvage, curative-intended, treatment options for these patients are limited, as they have been often considered medically inoperable already at initial diagnosis. Furthermore, only very limited data about lung re-irradiation exist. The experience with regards to lung re-irradiation is based exclusively on small retrospective series, more often applying SBRT only as a second course after first-line normofractionated treatments^9^, or only applying radiotherapy with palliative doses^10^. These studies could demonstrate that re-irradiation can be a reasonable option for palliation and that SBRT could also serve as a relatively effective salvage treatment after failure of normofractionated regimens^9–11^, although some authors observed excessive toxicity^12^. So, safety of such regimens remains unclear. Moreover, recent publications also investigated the application of repetitive SBRT-courses for lung malignancies, but all of these series used different definitions of re-irradiation and underlie several limitations^13,14^.

Aim of this multicentre retrospective study is to provide a comprehensive analysis of patterns of care, feasibility, safety and technical features of a second in-field SBRT course. The analysis will focus strictly on the rare cases planning target volume (PTV) overlap. Accumulated dose distributions of the first and second SBRT-course formed the basis for the analysis of dosimetric factors impacting safety and efficacy.

## Patients and methods

### Design, patients and data acquisition

This retrospective multicentric analysis was performed within the framework of the German Society of Radiation Oncology (DEGRO) working group for radiosurgery and stereotactic radiotherapy. The inclusion criteria for cases eligible for this analysis were: (1) thoracic malignancy (primary tumor or metastasis, regardless of histology) treated with SBRT in the past, (2) with a recurrence or second primary tumor with any distinct anatomic overlap of the initial primary target volumes (PTV) after rigid registration, (3) recurrence treated with a second course of SBRT, (4) where both SBRT-courses had to fulfill the criteria as defined SBRT by the DEGRO-group (a.o. ≤ 12 fractions, BED10 ≥ 50 Gy)^15^, (5) where the treatment plans and dosimetric parameters of both SBRT-courses are available in Digital Imaging and Communications in Medicine (DICOM) standard format. The patients were clinically followed for the first time 6–12 weeks and radiologically 3 months after each SBRT-treatment and both clinically and radiologically every 3 months thereafter using CT- or PET-CT-scans.

Eight centers in Austria, Germany and Switzerland contributed to this study. After the lead ethics committee approval at the University of Kiel (D 513/18) and subsequent approvals in all participating centers, the internal databases were examined for possible patients with SBRT re-irradiation to various thoracic tumors matching the above criteria. All patients included had signed an informed consent for treatment and general data acquisition. The data evaluated in this analysis was pseudonymized before central collection and evaluation.

Plans of both treatments including contours and doses were centrally collected in DICOM-format. All centers provided the following data, baseline information, toxicity according to CTCAE Version 5, technical data of both SBRT courses and data concerning tumor control, other oncological treatments and survival in a centrally developed database. Lesions within 2 cm of the proximal bronchial tree were defined as “central”^16^.

### Simulation and treatment

#### First SBRT course

Twelve patients were immobilized with the help of a vacuum bag, in other cases a WingSTEP (Elekta, eight patients) or a breast-board (1 patient) were utilized. Only four patients didn’t receive any special immobilization, whilst the remaining two were immobilized by a vacuum bag as well as the WingSTEP. Monte Carlo (17 patients) was the algorithm predominantly used for dose calculation, pencil beam/ ray tracing accounted for six patients, analytical anisotropic algorithm (AAA) for three and collapsed cone algorithm was utilized only once. Further information on the technical details of the first and second SBRT are summarized in supplementary table 1.

#### Second SBRT course

All but three patients were immobilized for the second SBRT course, for 13 patients a WingSTEP, for 10 a vacuum bag and for one a breast-board was used. The majority of dose-distributions were calculated using the Monte Carlo algorithm (21 patients) or collapsed cone algorithm in three patients.

18 Patients underwent 4D-CT simulation and the internal target volume (ITV) was applied in 13 patients. Image-guided radiotherapy (IGRT) using cone-beam computed tomography (CBCT) (19 patients) or stereoscopic kV imaging (eight patients) was used in all patients. Gated beam delivery was performed in 11 cases, robotic tracking in seven patients, beam delivery in breath hold in two patients and the remaining three patients were treated using abdominal compression; no active or passive motion compensation was used in only 4 patients.

### Extraction and summation of dosimetric parameters

**Figure 1:**
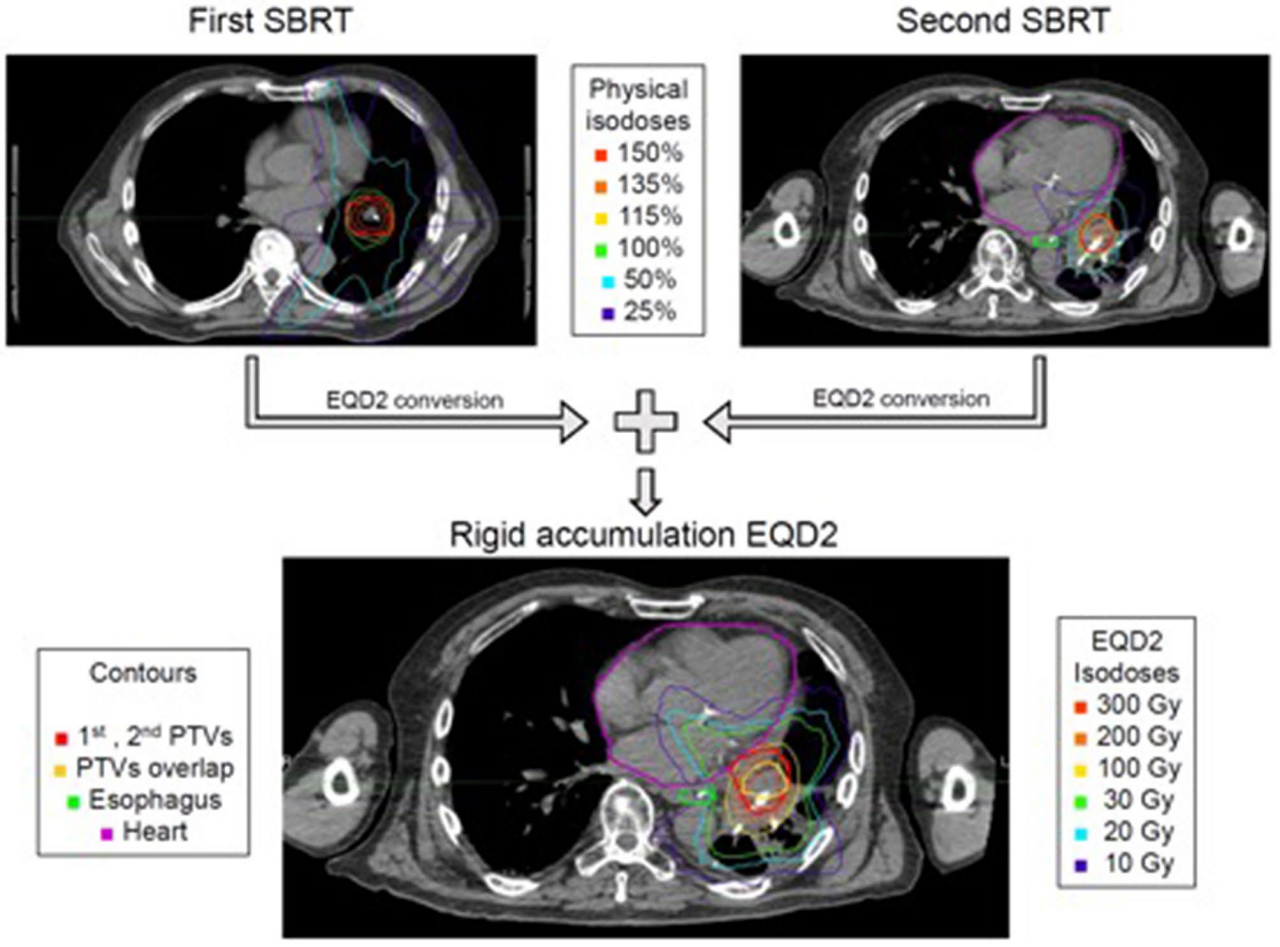
Exemplary case of the plan summation in MIM (version 6.9.2, MIM Software Inc., Cleveland, USA). The top row shows the first and second SBRT plans. The physical doses are normalized to 100% for the prescription to the PTV. Such doses are converted into EQD2 and rigidly accumulated onto the most recent CT. The result is shown at the bottom.

The dosimetric parameters were extracted using the software MIM (version 6.9.2, MIM Software Inc., Cleveland, USA). First, we converted each dose distribution into the corresponding 2 Gy equivalent dose (EQD2) distribution using the parameters α/β = 10 Gy (EQD2/10) for the tumor and α/β = 3 Gy (EQD2/3) for the surrounding normal tissue. Then, we performed a rigid registration of the planning CTs of the first and second SBRT courses. Overlap was evaluated on time resolved planning imaging in all motion phases available for all treatment techniques (e.g., ITV approach, gating, tracking, etc.). Overlap was defined as any overlap in any motion phase (supplementary figure 1). In presence of significant anatomical changes, rigid registration was performed on a smaller ROI centered on the re-irradiated target volume. The structures and the EQD2 dose distributions from the first SBRT were rigidly propagated onto the most recent planning CT. We used the Boolean operator “*and”* to define the geometrical overlap between the first- and second-course PTV, which we extracted the volume of. After summing the two EQD2 dose distributions, we extracted the mean, point maximum, D1cc and D0.1 cc EQD2 values for the relevant organs. We chose to extract robust parameters, which are computed only locally in the proximity of the tumor: for the lungs, we extracted: V10Gy, V20Gy, V30Gy, V100Gy and V200Gy in cubic centimeters (cm^3^). An example of a plan summation is illustrated in Fig. 1.

### Statistical analysis

Progression was considered any primary tumor site, local or distant progression which occurred after the beginning of the second SBRT course identified either through CT or PET scan. Overall survival (OS) was defined as time between the start of the 2nd treatment to death from any cause. For both local and distant control rate, we established the start of the 2nd SBRT course as the baseline. Local control rate (LCR) was determined between baseline and any unequivocal radiological proof of local progression as event. Furthermore, distant control rate (DCR) was defined as any radiological evidence of progression of existing non-target lesions (RECIST criteria), as well as appearance of one or more new lesions. For the estimation of local and distant control, as well as survival probabilities, we utilized the Kaplan–Meier method. Log-rank test was implemented for univariate analyses. Statistical significance was defined at *p* < 0.05. We used IBM SPSS Statistics (version 25, IBM, Armonk, USA) for all statistical analyses. The median and the corresponding range of values were calculated for all variables of interest.

### Ethics approval

All procedures performed in this study involving human participants were in accordance with the ethical standards of the institutional and national research committee and with the 1964 Helsinki declaration and its later amendments or comparable ethical standards.

## Results

### Patient and tumor characteristics

We evaluated a total of 27 patients who fulfilled the study inclusion criteria, treated from 2009 (oldest first course) to 2020 (latest second course). Out of these 27 cases, nine were female (33.3%) and 18 were male. The age at 1st SBRT ranged from 34 to 88 years with a median of 71 years. Further patient-, tumor-, and treatment-related characteristics are summarized in Table 1. In 12 patients, the histology of the primary tumor was consistent with a non-small-cell lung cancer (NSCLC), whereas eight other patients were treated for pulmonary metastases of colorectal cancer, melanoma and breast cancer contributed in one patient, and four other primary tumors. In 11 patients, the target lesion was the primary tumor, in the other 16 patients, metastasis directed SBRT was performed, with 10 of the lesions in a central location. Driver mutations were identified in four patients, in 10 cases the mutational analysis was not indicated and in an additional 13 cases the mutation profile was negative for driver mutations. The majority of 63% of patients presented in stages I-II at baseline (before any treatment), 18.5% at stage III and another 18.5% were metastasized at baseline. Thirteen of the patients underwent a multimodal initial treatment (i.e. primary treatment at first diagnosis of malignancy) whilst 14 were initially treated with one single modality: surgery (11 patients), chemotherapy (12 patients), radiation (15 patients), targeted therapy (two patients).

**Table 1:** Clinical and treatment-related characteristics, including initial treatment and stage at initial diagnosis.

Characteristics		Value (% or range)
Age at 1st SBRT	Median in years	71 (34–88)
Gender		
Male		18 (66.7)
Female		9 (33.3)
**Primary tumor**		
Histology		
NSCLC		12 (44.4)
CRC		8 (29.6)
Breast cancer		1 (3.7)
Melanoma		1 (3.7)
Others		4 (14.8)
Initial stage at diagnosis		
Stage I–II		17 (63)
Stage III		5 (18.5)
Metastases at baseline		5 (18.5)
Initial treatment at first diagnosis of malignancy^*a*^		
Surgery		11 (40.7)
Chemotherapy		12 (44.4)
Radiation		15 (55.5)
Targeted therapy		2 (7.4)
Driver mutations		
Yes		4 (14.8)
EGFR: Deletion in Exon 19	NSCLC	1 (3.7)
KRAS: Codon 12	CRC	1 (3.7)
BRAF: pD594G	CRC	1 (3.7)
Hormone-receptor positive	Breast cancer	1 (3.7)
No		13 (48.1)
Test not indicated		10 (37)
**Target lesion**		
Primary tumor		11 (40.7)
Metastasis		16 (59.3)
Localization		
Central		10 (37)
Peripheral		17 (63)
Confirmation of recurrence		
PET		13 (48.1)
SUV of recurrence	Median	8.6 (1.8–22.6)
CT Thorax		12 (44.4)
Biopsy		2 (7.4)
Primary controlled at time of 2nd SBRT		
Yes		11 (40.7)
No		16 (59.3)

^a^Some patients had more than one initial treatment.

SBRT, stereotactic body radiation therapy; NSCLC, non-small cell lung cancer; CRC, colorectal cancer; PET, positron emission tomography; SUV, standardized uptake value; CT, computed tomography; EGFR, epidermal growth factor receptor; KRAS, Kirsten rat sarcoma viral oncogene; BRAF, Serine/Threonine-Kinase B-Raf.

### Treatment characteristics

#### First SBRT-course

Out of the evaluated patients, 17 presented with a singular lesion (primary or metastasis) for the first SBRT, the remaining 10 patients had either one or two metastases (six patients) or more than three metastases (four patients). In the first SBRT course a median of 5 fractions (range: 1–11) with a median prescribed dose of 38.5 Gy (D98%, range: 20–85.8 Gy) was applied to a median planning target volume (PTV) of 35 cm^3^ (range: 6.3–505.5 cm^3^). The median prescription isodose line was at 65% (range: 58–100%). Five patients were subjected to systemic therapy during the first SBRT course. The data of the treatment characteristics for the first and second SBRT are summarized in Table 2.

**Table 2:** Treatment characteristics 1st and 2nd SBRT.

Characteristics		Value (% or range)
**1st SBRT**		
Irradiated lesion = singular lesion		
Yes		17 (63%)
No		10 (37%)
Stage at time of 1st SBRT		
M0		9 (33.3%)
M1		18 (66.7%)
Number of (additional) metastases		
None		17 (63%)
1 or 2		5 (18.5%)
3 or more		5 (18.5%)
Number of affected organs		
None		21 (77.8%)
1 or 2		4 (14.8%)
3 or 4		2 (7.4%)
Number of fractions	Median (range)	5 (1–11)
5		9 (33.3%)
3		6 (22.2%)
1		4 (14.8%)
Other		8 (29.6%)
PTV	Median in cm^3^ (range)	35 (6.3–505.5)
Prescribed dose	Median in Gy (range)	38.5 (20–85.8)
Enclosing isodose	Median % (range)	65 (58–100)
Systemic therapy during SBRT		
Yes		5 (18.5%)
Nivolumab		2 (7.4%)
Cisplatin, Etoposid		1 (3.7%)
Erlotinib		1 (3.7%)
Gemcitabine, Abraxane		1 (3.7%)
No		22 (81.5%)
**Time intervals between SBRTs**	Median in months (range)	20.2 (3–89)
**2nd SBRT**		
Irradiated lesion = singular lesion		
Yes		18 (66.7%)
No		9 (33.3%)
Stage at time of 2nd SBRT		
M0		6 (22.2%)
M1		21 (77.8%)
Number of (additional) metastases		
None		18 (66.7%)
1		4 (14.8%)
2 or 3		4 (14.8%)
5		1 (3.7%)
KPS 2nd SBRT	Median % (range)	80 (60–100)
Number of fractions	Median (range)	5 (1–12)
5		10 (37%)
3		4 (14.8%)
8		4 (14.8%)
Other		9 (33.3%)
PTV	Median in cm^3^ (range)	29.5 (5.32–559.3)
Prescribed dose	Median in Gy (range)	40 (19–66)
Enclosing isodose	Median % (range)	65 (55–100)
Systemic therapy during Re-SBRT		
Yes		7 (25.9%)
Taxol, anti-TIM3, anti-PD1		1 (3.7)
EGFR-Inhibitor (AZD9291)		1 (3.7)
Nivolumab		1 (3.7)
4 cycles FLO^a^		1 (3.7)
5-FU, Irinotecan		1 (3.7)
Erlotinib		1 (3.7)
Folfirinox		1 (3.7)
No		20 (74.1%)

^a^Administered 1 week post Radiation for a synchronous stomach cancer.

PTV, planned target volume; KPS, Karnofsky performance status; Anti-TIM3, anti-T-cell immunoglobulin and mucin domain-3; Anti-PD1, anti-programmed cell death protein-1; EGFR, epidermal growth factor receptor; FLO, 5-Fluorouracil, Folinic acid and Oxaliplatin; Folfirinox, Fluorouracil, Folic acid, Irinotecan, Oxaliplatin.

#### Second SBRT-course:

The median time interval between the first and second SBRT course was 20.2 months, ranging between 3–89 months. At the time of 2nd SBRT, 11 patients presented in a very good general condition with a Karnofsky Performance Status (KPS) 90%-100%; nine patients were characterized by a KPS of maximum 70%.

The re-irradiation PTV was median 29.5 cm^3^ (range, 5.32–559.3 cm^3^) and the median prescribed dose was 40 Gy (range, 19–66 Gy), which was prescribed to the PTV enclosing isodose of median 65% (range, 55–100%). The median number of SBRT fractions was five (range, 1–12). During the 2nd SBRT 7 patients received systemic therapy. The detailed characteristics of the 2nd SBRT are also listed in Table 2 and suppl. table 1.

### Accumulated SBRT plans

Cumulative dosimetric data is summarized in Table 3. The median PTV overlap of the two SBRT courses was 22 cm^3^ (range: 0.1–184.5 cm^3^). The median cumulative PTV EQD2/10 maximum dose (Dmax) was 270.04 Gy (range: 78.3–619.9 Gy), the median cumulative mean lung dose in EQD2/3 was 9.1 Gy (range: 2.0–19.6 Gy) and the median cumulative mean lung dose EQD2/3 of the ipsilateral lung was 14.8 Gy (4.0–30.4 Gy). 0.1 cm^3^ of the PTV received a median EQD2/10 dose of 262.9 Gy and 1 cm^3^ received a median of 248.6 Gy. The median V100Gy (EQD2) of the summed plans amounted 83.8 cm^3^, and the median V200Gy and V300Gy 9 cm^3^ and 0 cm^3^ respectively. Furthermore, the cumulative median lung volume receiving an EQD2 dose of 10 Gy (V10) was 624.9 cm^3^ (range: 10.6–2350.9 cm^3^), the median volume receiving 20 Gy (V20) and 30 Gy (V30) were 371.8 cm^3^ (range, 20.7–1543.4) and 277.4 cm^3^ (range: 10.8–975.8 cm^3^), respectively. In the summed plans 10% of the lung volume received a median of 20.7 Gy (range: 4.83–50.20 Gy), respectively 20% and 30% of the lung volume obtained 9.25 Gy (range: 2.4–32.9 Gy) and 4.17 Gy (range: 1.4–25.8 Gy). The V100Gy, V200Gy and V300Gy for both lungs summed up respectively to 51.95 cm^3^, 2.3 cm^3^ and 0 cm^3^. The summed EQD2 to 1000 cm^3^ and to 1500 cm^3^ amounted to 4.39 Gy (range, 0.65–29.53 Gy) and 1.81 Gy (0.27–20.85 Gy) respectively.

**Table 3:** Dosimetric data of the combined plans (also with respect to TG101 thresholds).

		Median value (range)
PTV overlap	Median in cm^3^ (range)	22 (0.1–184.50)
Sum EQD2 Mean	In Gy	
Lungs both		9.1 (2.02–19.62)
Affected lung		14.8 (3.98–30.39)
Sum EQD2 max	In Gy	
PTV		270.04 (78.33–619.87)
Lung		
1000 cm^3^		4.39 (0.65–29.53)
1500 cm^3^		1.81 (0.27–20.85)
Heart		33.8 (0.07–357.25)
0.1 cm^3^		30.85 (0.72–340.78)
1 cm^3^		25.28 (0.69–298.95)
5 cm^3^		15.98 (0.55–130.73)
Esophagus		21.86 (4.94–94.38)
0.1 cm^3^		19.25 (4.51–83.50)
1 cm^3^		16.79 (3.41–74.49)
5 cm^3^		12.13 (0.49–69.58)
Thorax/Ribs		118.03 (64.11- 235.54)
0.1 cm^3^		< 1 Gy
30 cm^3^		< 1 Gy (max. 103.57)
Proximal bronchial tree^a^		
4 cm^3^		26.72 (0.71–91.17)
Sum Lungs EQD2	In Gy	
D10%		20.7 (4.83–50.20)
D20%		9.25 (2.40–32.90)
D30%		4.17 (1.40–25.84)
Sum Lungs EQD2	In cm^3^	
V10 Gy		624.9 (10.60–2350.90)
V20 Gy		371.8 (20.70–1543.40)
V30 Gy		277.4 (10.80–975.80)
V100 Gy		51.95 (0–152.40)
V200 Gy		2.3 (0–51.45)

^a^Evaluated only for 19 patients with central localization or any relevant dose.

PTV, planning target volume; Dx%, dose received by x% of PTV; VxGy, volume receiving x Gy; EQD2, total equivalent dose in 2 Gy fractions.

Regarding other organs at risk, the median cumulative heart EQD2/3 Dmax was 33.8 Gy (range: 0.1–357.3 Gy), with 1 cm^3^ and 5 cm^3^ receiving 25.3 Gy and 15.98 Gy respectively. There were only three patients with a heart volume receiving an EQD2 V100Gy: 2.38, 3.74 and 23.82 cm^3^. The median cumulative esophagus EQD2/3 Dmax was 21.9 Gy (range: 4.94–94.4 Gy). A median EQD2 of 12.13 Gy was applied to 5 cm^3^ of esophagus (range: 0.49–69.58). A cumulative Dmax > 100 Gy EQD2 for the esophagus was not observed. Regarding the rib cage/thorax the median cumulative EQD2/3 Dmax in the five patients with tumor directly adjacent to the ribs was 118.3 Gy (range: 64.1–235.5 Gy). As the chest wall was in close proximity of the PTV only in these five cases no median dose was calculated for 0.1 cm^3^ and 1 cm^3^ and there was only a single case with a relevant V100Gy of 33.0 cm^3^. The maximum D30 cm^3^ observed for the chest wall was 103.57 Gy. Finally, the cumulative dose to the proximal bronchial tree was calculated only in 19 cases with either central location or any relevant dose to that structure: for these cases 4 cm^3^ of proximal bronchus/trachea received a median EQD2 of 26.72 Gy (range: 0.71–91.17).

### Oncological endpoints and toxicity

#### Oncological endpoints

**Figure 2:**
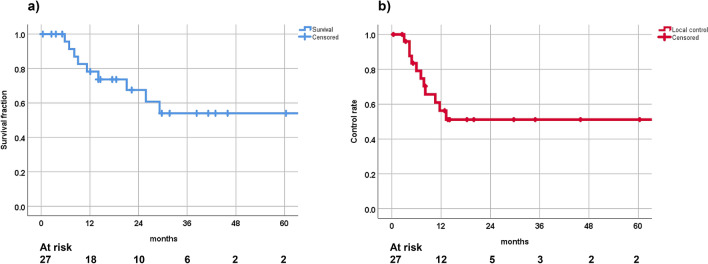
(**a**) Overall survival and (**b**) local control rate after the 2nd SBRT.

After a median follow up of 17.52 months (mean 22.54, range, 0.4–76.2) the 1-year and 2-years OS was 78.3% and 67.5%, respectively. The DCR was 73.8% after one year and 30.9% after two years. Local control rate at 1-year and 2-years was 70.3% and 51.1%, respectively. Altogether, 11 out of 27 patients experienced a local recurrence. For the Kaplan–Meier curves for OS and freedom from local progression see Figs. 2a,b.

Exploratory univariate analyses were performed for the oncological endpoints OS, LCR, DCR, considering the small sample size. Age, sex, tumor histology, primary versus metastatic lesion, tumor localization, prescribed and maximum ReSBRT-dose, summed Dmax, further metastases and intervall between SBRT courses were used as independent variables. With regard to the OS, a higher dose for the 2nd treatment course (≥ median vs lower) significantly correlated with a longer OS (*p* = 0.005). Moreover, a controlled primary at the time of 2nd SBRT correlated with improved OS (*p* = 0.055).

A peripheral anatomical localization was associated with a significantly better local control compared to a central one (*p* = 0.013). The same was true only for a higher or equal than median dose of the 2nd SBRT-course (*p* = 0.055). Primary lesions were not controlled significantly better or worse after re-SBRT compared to metastatic lesions (*p* = 0.9). The length of the time interval between the 2 SBRTs (≥ median interval vs shorter) and the absence of metastases correlated significantly with a longer DCR (*p* = 0.033, respectively *p* = 0.023).

**Figure 3:**
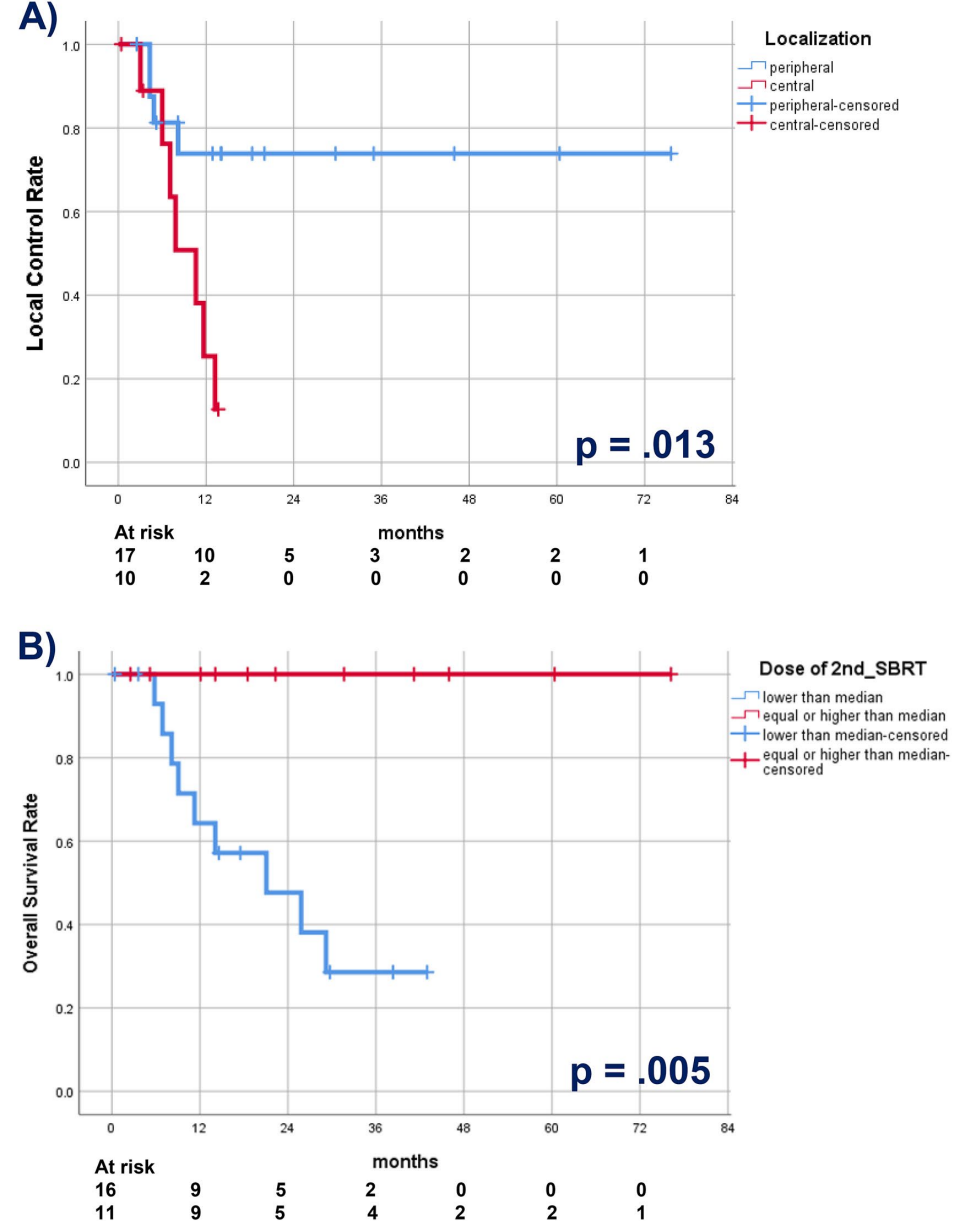
Kaplan–Meier curves demonstrating the significant impact of (**A**) tumor localization on local control rate and (**B**) dose of the 2nd SBRT course on survival.

The Kaplan–Meier curves demonstrating the parameters with significant influence on OS and LCR are depicted as Fig. 3A,B. No other parameter with significant influence could be found for any of the oncological endpoints.

#### Toxicity

None of the patients experienced any toxicity higher than grade 2 after the second SBRT-course. Three out of 27 patients (11.1%) presented with radiological signs consistent with grade 1 pneumonitis (no symptoms or very mild symptoms), while only one person showed symptoms of grade 2 pneumonitis requiring medication; the remaining 23 patients tolerated the re-irradiation without showing any signs of pneumonitis or other sequela. Especially, no cases of rib or soft tissue necrosis or esophageal/bronchial fistula were observed. Moreover, there were no treatment-related hospitalizations or deaths. Due to the low rates of overall toxicity no valid predictive factors for pneumonitis or other adverse events could be found. However, the only patient with grade II pneumonitis after the second SBRT was the only one receiving concomitant immunotherapy, namely Nivolumab. Toxicity is summarized in Table 4.

**Table 4:** Toxicity after 2nd SBRT.

Toxicity	Value (% or range)
Pneumonitis after 2nd SBRT	
Grade 0	23 (85.1)
Grade 1	3 (11.1)
Grade 2	1 (3.7)
Other toxicity > 2 after 2nd SBRT	0

## Discussion

The present study analyzed the feasibility and efficacy of a second in-field SBRT for thoracic malignancies, with a focus on analysis of accumulated dose distributions for more accurate description of dosimetry. In our retrospective multi-institutional analysis, a second course of SBRT with volume overlap appeared feasible without unexpected toxicity, resulting in reasonable disease control rates.

Several studies investigating repetitive thoracic irradiation have been published so far. Drodge et al. conducted a review of 13 publications and 379 cases gathered over approximately 3 decades, with most patients being symptomatic at the time of the second course and with the median (palliative) dose used not exceeding an EQD2 of 36 Gy^10^. Nevertheless, this low-dose re-irradiation (ReRT) resulted in excellent control of the symptom burden with manifestations like hemoptysis, coughing and pain showing improvement in the vast majority of patients. Although the toxicity-rates in the older studies included in the review were relatively low, treatment-related deaths amounted to no fewer than 1.6%. One of these studies, applying a “full-dose-course” of ReRT with EQD2 of 60 Gy resulted in an even higher death-rate with 3/24 patients expiring. Similarly, exceeding rates of grade 5 toxicity (6/52 patients) were observed in a multi-institutional prospective trial investigating thoracic ReRT for non-small-cell lung cancer (NSCLC) using proton beams and very similar doses of median 66.6 Gy^17^. Treated target volume was the most important predictor of severe toxicity. Patients with volumes < 41 cm^3^ experienced significantly less sequela of grade 3 or higher. Furthermore, the study had been closed early for recruitment of patients with clinical target volume (CTV) > 250 cm^3^ and the authors concluded that thoracic ReRT is feasible for CTV smaller than 250 cm^3^, but associated with considerable side effects. Following these data of normofractionated ReRT, a series of more recent studies with mostly low patient numbers (8–39 patients) investigated the application of SBRT as salvage treatment to mostly small recurrences after normofractionated first RT-course^11,18–20^. The same is true for the largest cohort published so far by Liu et al. consisting of 72 patients, treated after a median interval of 21 months with 50 Gy in 4 fractions and with approximately 20% of the patients developing severe pneumonitis^21^. Other studies included mostly non-overlapping volumes and ReRT was defined as overlap of the 25%- or 30%-isodose^22^ or within 1 cm of the primary tumor^13^. Altogether, while taking into account the strong heterogeneity of these data, salvage SBRT for lung malignancies resulted in local control rates of 52–92% and grade 3–5 toxicity of 0–30%^9^.

Few studies analysed the rare scenario, where a second SBRT course was applied for in-field local recurrences or second primaries in the lung (supplementary table 2)^12–14,23^. In the absence of a common definition of “re-irradiation” in these studies, only Peulen et al. used a definition based upon target volume overlap as in the present study. However, as the study of Peulen et al. was conducted over 15 years ago, with treatments originating even from the early 90 s, only three-dimensional measurements were conducted in order to define this overlap. In the present study, both plans were centrally collected for all cases as described under “methods” and the summed dosimetric and volumetric parameters for PTV and organs at risk (OAR), including EQD2 calculations, were calculated after rigid registration, consideration of time-resolved planning and evaluation in the software MIM. The selection of cases in this analysis was based on overlapping PTVs, where in fact the 55–100% isodose lines overlapped between the two SBRT treatments; i.e. a higher isodose than in the previous publications. Yet, the results are comparable to these publications, which included similar or lower case-numbers (10–31 patients), without higher toxicity rates. The time interval between both SBRT courses was similar with one to two years in all studies, such being comparable to the median interval of 20.2 months observed for the present cohort. The exact volume size treated, either GTV nor PTV, is not clearly stated in all of the previous publications (some of them prefer tumor diameter). Yet, if one computes a spherical tumor/PTV only two of the four studies (Peulen et al., Ogawa et al.) treated somewhat larger volumes, at least when considering the median.

Regarding feasibility and toxicity, only Peulen et al. reported cases of severe toxicity, even resulting in fatal bleeding in 3 cases, all of them being centrally located^12^. Importantly, our study was the only other one including > 30% centrally located cases, without similar deathly events being observed. The limited toxicity in most studies is in line with the results presented here, underlining once more the feasibility of salvage-SBRT in the thorax, even after previous SBRT. Previous studies in brain radiosurgery/ stereotactic radiotherapy seem to confirm the relative safety of high-dose, in-field, ReRT^24–26^. It appears that re-treating a small volume twice, might result in a lower risk of complications like pneumonitis compared to repeated courses over the whole lung volume. Especially, if the assumption is made that this volume is already fibrotic after the first high-dose course. Of course, additional risks have to be taken into account when applying re-irradiation in immediate proximity of vulnerable serial organs at risk, such as the esophagus or the central bronchial tree.

Notwithstanding the limitations of the very heterogeneous cases with respect to histology, stage and dose/ fractionation regimens presented here, as well as in the other cited studies we could observe a relative good efficacy of a thoracic Re-SBRT resulting in one-year control rates of slightly lower than 80% and a considerable percentage of longer-term survivors of over 50%, approximately 60% of them with locally controlled tumor. Although these results might be further improved with more careful patient selection (peripherally located, smaller tumors) and dose escalation, they appear reasonable for this difficult to treat and sometimes heavily pretreated collective, where the Re-SBRT was often indicated due to the lack of alternatives. Moreover, some of the fractionation and dose regimens applied here, although matching the SBRT-definition of the study group^15^, are internationally recognized as “moderately” and not as “ultra-” hypofractionated, which might explain the somewhat lower local control rates compared to other series. Interestingly, even in this relatively small cohort analyzed here, we could observe a significant dose–response relationship with higher Re-SBRT doses correlating with improved survival- and local control rates. Although, the results could be biased due to the retrospective character of the study with larger or centrally located tumors tending to receive lower doses, a dose–response relationship after primary SBRT has been well described for NSCLC, pulmonary metastases and other tumors^27,28^ and this is probably also the case in the recurrent situation. As the vast majority of patients will ultimately develop distant progression (70% after two years in our study), adding modern, more effective, systemic treatment^29^ in order to control systemic disease could also facilitate long-term survival and in the near future, local control could become even more important.

There are several limitations and drawbacks of this study: first of all, the limited number of cases, secondly the great heterogeneity of the cases included, third the not internationally accepted definition of SBRT used in this cohort and finally the retrospective design. Especially the heterogeneity in terms of histology, stages and initial treatment and the definition of SBRT, which is not consistent with the north-American practice could hamper generalizability of the observations. Both “radioresistant” and “radiosensitive” histologies were included here and some of the regimens used applied a relatively low dose compared to international standards. These parameters can clearly affect tumor control and toxicity. Moreover, both patients with single lesions and some with additional metastases were included, which makes safe conclusions regarding OS and possible survival benefits difficult. Nevertheless, this study evaluates a second, exclusively in-field-course of SBRT for thoracic malignancies, in a multicentre setting, including central detailed evaluation and summation of both treatment plans.

## Conclusion

A second course of thoracic SBRT for overlapping volumes is feasible and associated with low toxicity rates for well selected patients. The tumor control rates are reasonable and strongly depend on the applied dose.

## Supplementary Information

Supplementary Information 1.

Supplementary Information 2.

Supplementary Information 3.

## Data Availability

All data will be made available upon request.
