# Supplementary material for: In-field stereotactic body radiotherapy (SBRT) reirradiation for pulmonary malignancies as a multicentre analysis of the German Society of Radiation Oncology (DEGRO)
Source: Sci Rep. 2021 Feb 25;11:4590. doi: 10.1038/s41598-021-83210-3 (PMC7907095; doi:10.1038/s41598-021-83210-3)
Supplement: Supplementary file 1 — Supplementary Information 1. [file 41598_2021_83210_MOESM1_ESM.docx]

Suppl. Tbl.1: Technical characteristics of the SBRT-courses

| **1^st^ SBRT** |  | **(%)** |
| --- | --- | --- |
| Additional Immobilisation * |  |  |
| None |  | 4 (14.8) |
| Vacuum bag |  | 12 (44.4) |
| WingSTEP |  | 8 (29.6) |
| Breast-board |  | 1 (3.7) |
| Vacuum bag + WingSTEP |  | 2 (7.4) |
| Algorithm |  |  |
| Pencil Beam/ Ray Tracing |  | 6 (22.2) |
| Collapsed Cone |  | 1 (3.7) |
| AAA (analytical anisotropic algorithm) |  | 3 (11.1) |
| Monte Carlo |  | 17 (63) |
|  |  |  |
| **2^nd^ SBRT** |  |  |
| Platform |  |  |
| Linac |  | 20 (74) |
| Cyberknife |  | 7 (26) |
| Additional Immobilisation |  |  |
| None |  | 3 (11.1) |
| Vacuum bag |  | 10 (37) |
| WingSTEP |  | 13 (48.1) |
| Breast-board |  | 1 (3.7) |
| Algorithm |  |  |
| Pencil Beam/ Ray Tracing |  | 1 (3.7) |
| Collapsed Cone |  | 3 (11.1) |
| AAA (analytical anisotropic algorithm) |  | 2 (7.4) |
| Monte Carlo |  | 21 (77.8) |
| Breath Management |  |  |
| None |  | 4 (14.8) |
| Abdominal compression |  | 3 (11.1) |
| Breath hold |  | 2 (7.4) |
| Gating |  | 11 (40.7) |
| Tracking |  | 7 (25.9) |
| IGRT |  |  |
| CBCT |  | 19 (70.4) |
| Stereoscopic kV |  | 8 (29.6) |
| 4D CT |  |  |
| Yes |  | 18 (66.7) |
| No |  | 9 (33.3) |
| ITV |  |  |
| Yes |  | 13 (48.1) |
| No |  | 14 (51.8) |
| GTV | Median in cm^3^ (range) | 11.7 (0.48-247.6) |
| *For some of the patients none of the above methods were used for others more than one SBRT, stereotactic body radiation therapy; AAA, analytical anisotropic algorithm; IGRT, image guided radiation therapy; CBCT, cone beam computed tomography, kV, kilo-voltage; CT, computed tomography; ITV, internal target volume, GTV, gross tumor volume | | |
