# Supplementary material for: In-field stereotactic body radiotherapy (SBRT) reirradiation for pulmonary malignancies as a multicentre analysis of the German Society of Radiation Oncology (DEGRO)
Source: Sci Rep. 2021 Feb 25;11:4590. doi: 10.1038/s41598-021-83210-3 (PMC7907095; doi:10.1038/s41598-021-83210-3)
Supplement: Supplementary file 2 — Supplementary Information 2. [file 41598_2021_83210_MOESM2_ESM.docx]

Supplementary table. 2: Overview of studies reporting on Re-SBRT

|  | Hearn et al. (13) | Peulen et al. (12) | Ogawa et al. (14) | Kennedy et al. (23) | present study |
| --- | --- | --- | --- | --- | --- |
| **Definition Re-SBRT** | Overlap occurring within 1 cm of the planning target volume (PTV) | More than 50% overlap of the planning target volume (PTV) | “in-field relapse” | Lesions within 1cm of the original PTV and/or new primaries having overlap of at least the 25% prescription isodose | Any anatomic overlap of the planning target volumes (PTV) |
| **Number of patients (n)** | 10 | 29 | 31 | 21 | 27 |
| **Tumor size in cm, or PTV 2^nd^ SBRT in cm^3^** | *Tumor diameter:*  3.4 cm (1.7-4.8) | 76 (16–355) | 69.8 (10.2–149) | *Tumor diameter:*  1.4 cm (1-2.5) | 29.5 (5.32-559.3) |
| **Side effects** | grade >II: 0% | grade III-IV: 27.6%  grade V (bleeding): 10.3% | grade II pneumonitis: 12.9%  grade >II: 0% | grade II pneumonitis: 10%, grade II chest wall: 19%  grade >II: 0% | grade II pneumonitis: 3.7%  grade >II: 0% |
| **OS/ LCR** | LCR: 60% | 1-OS: 59% | 1-y-OS: 82%* 1-y-LCR: 78%*  3-y-OS: 36% 3-y-LCR: 53% | 1-y-OS: 82%*    2-y-OS: 68% 2-y-LCR: 81% | 1-y-OS: 78.3% 1-y-LCR: 70.3%  2-y-OS: 67.5% 2-y-LCR: 51.1% |

* estimated from curve
