# Supplementary material for: In-field stereotactic body radiotherapy (SBRT) reirradiation for pulmonary malignancies as a multicentre analysis of the German Society of Radiation Oncology (DEGRO)
Source: Sci Rep. 2021 Feb 25;11:4590. doi: 10.1038/s41598-021-83210-3 (PMC7907095; doi:10.1038/s41598-021-83210-3)

**Supplementary figure 1** two examples of detecting the PTV-overlap with the help of 4D-CT-scans as analyzed in MIM (version 6.9.2, MIM Software Inc., Cleveland, USA)

1. **Volume PTVs overlap = 0.4cc**

Overlap as seen in the 4DCT_AVG registration


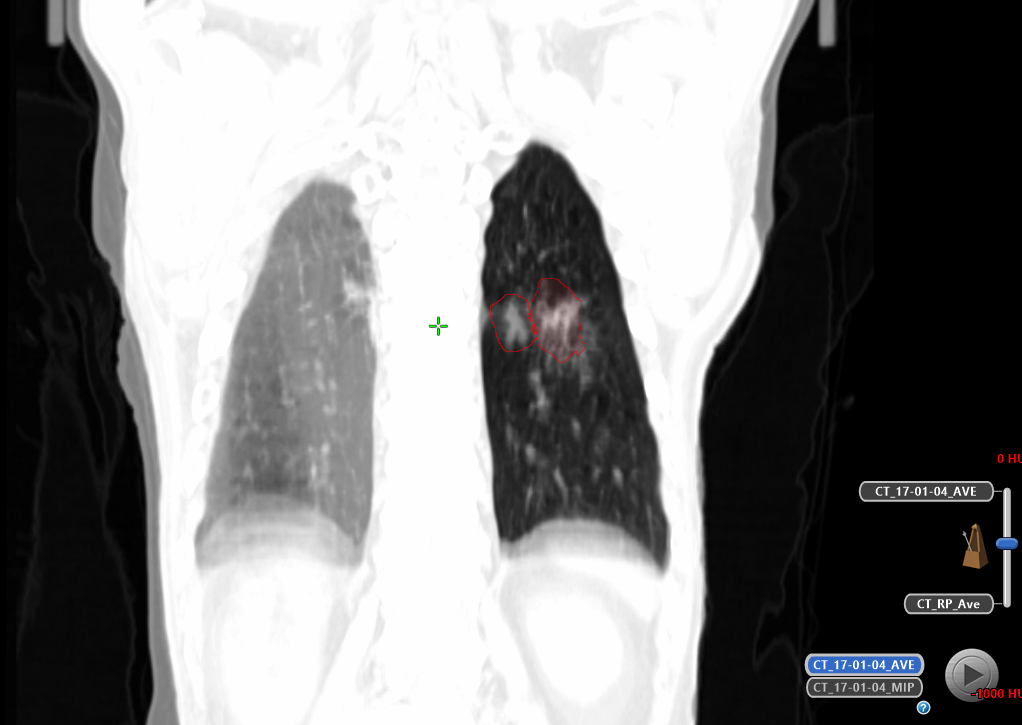


PTV 2nd SBRT

PTV 1st SBRT

Blending of the single phases of the 4DCT

Minimal overlap Maximal overlap


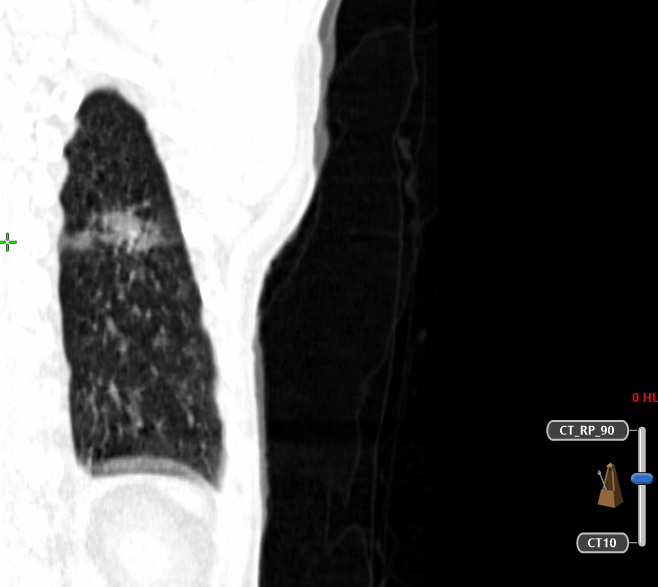

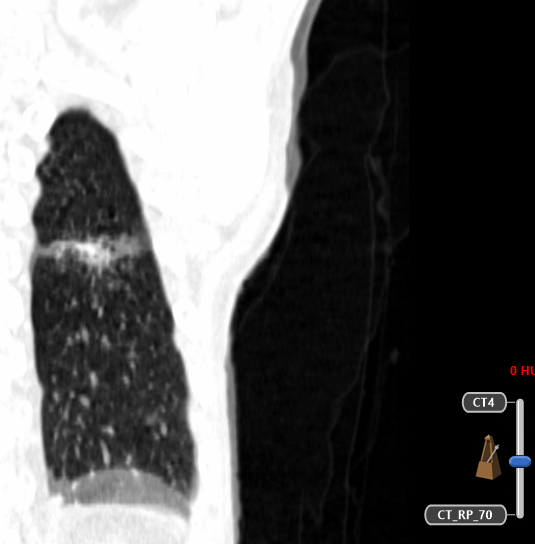


1. **Volume PTVs overlap = 118.5cc**

Overlap as seen in the 4DCT_AVG registration


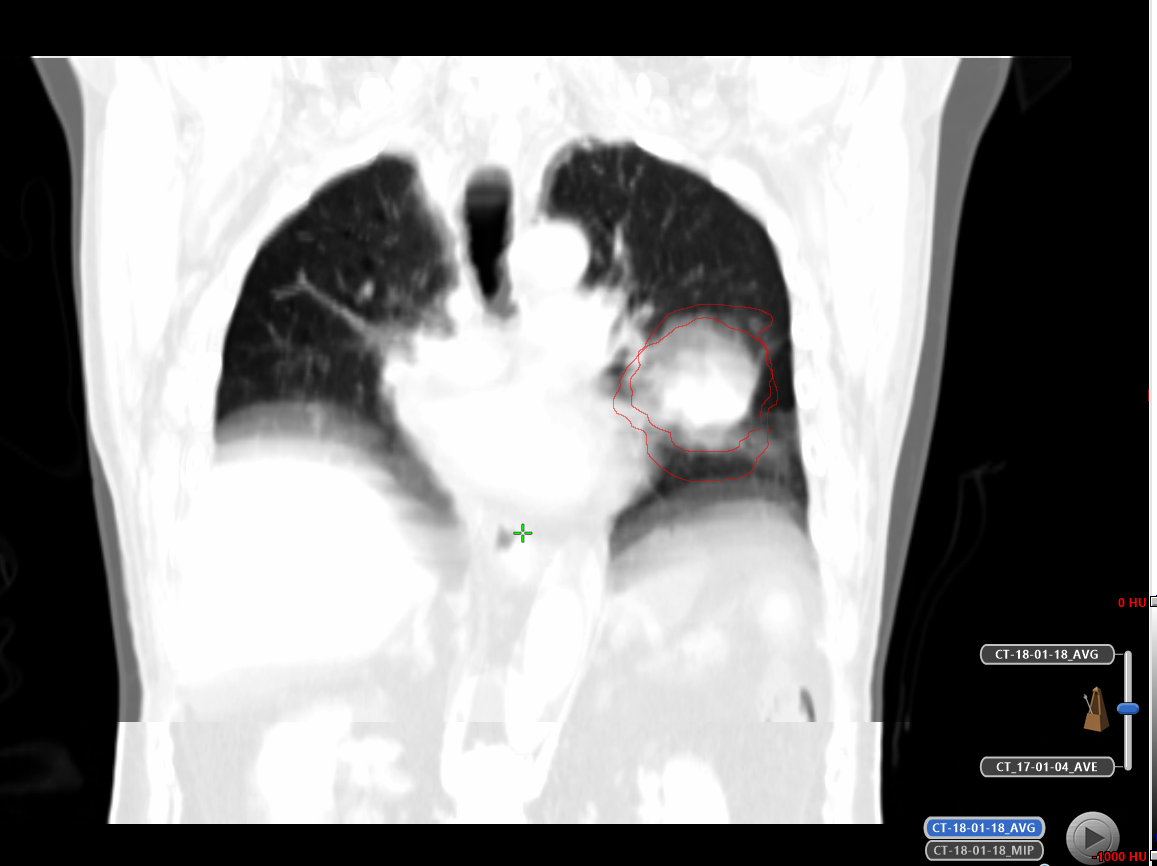


PTV 2nd SBRT

PTV 1st SBRT

Blending of the single phases of the 4DCT

Minimal overlap Maximal overlap


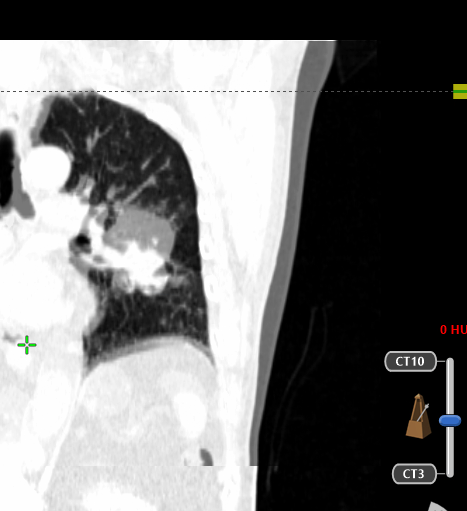

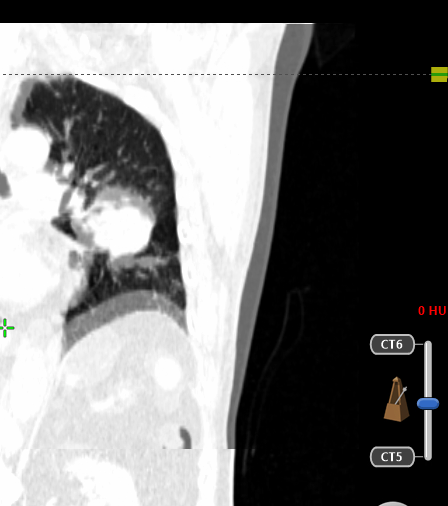

Supplement: Supplementary file 3 — Supplementary Information 3. [file 41598_2021_83210_MOESM3_ESM.docx]
